# Preservation and Protection of Cultural Heritage: Vibration Monitoring and Seismic Vulnerability of the Ruins of Carmo Convent (Lisbon)

**DOI:** 10.3390/s24186095

**Published:** 2024-09-20

**Authors:** Nuno Mendes, Nicoletta Bianchini, Georgios Karanikoloudis, Anna Blyth, Jacopo Scacco, Luis Gerardo Flores Salazar, Cassie Cullimore, Lavina Jain

**Affiliations:** ISISE, ARISE, Department of Civil Engineering, University of Minho, 4800-058 Guimarães, Portugal; nicoletta.bianchini@gmail.com (N.B.); karanikoloudis@hotmail.com (G.K.); balzac.js@gmail.com (J.S.); gsalazar@fe.up.pt (L.G.F.S.); cassiecullimore@cmail.carleton.ca (C.C.); gcad10114@gmail.com (L.J.)

**Keywords:** historical construction, masonry structures, metro-induced vibrations, structural health monitoring, finite element method, discrete element method

## Abstract

Preservation of cultural heritage sites is of paramount importance. The ruins of Carmo Convent in Lisbon stand as a poignant reminder of the city’s rich history, but challenges regarding structural integrity and safety are present in a highly populated and touristic area. In this paper, a comprehensive study of the Carmo Convent is presented, focused on non-destructive testing (NDT), structural health monitoring (SHM) and numerical modelling. Given its state of ruin and historical relevance, the study relied heavily on NDT. Additionally, a metro line passing underneath the convent raised concerns regarding potential hazards from induced vibrations. Thus, metro vibration monitoring (MVM) was implemented to assess the impact of induced vibrations on the structure. One of the challenges was the scarcity of standards specific to historic structures. However, through a combination of finite element method (FEM) and discrete element method (DEM) numerical modelling, valuable insights into the current condition of the structure were obtained. MVM revealed that the maximum velocity induced by metro activities remained within safe limits, indicating minimal impact. These results not only provide crucial information on structural preservation but also empower stakeholders to make informed decisions regarding the implementation of protective measures.

## 1. Introduction

The preservation of cultural heritage sites is a critical endeavour, essential for maintaining the historical legacy and cultural identity of societies. The Carmo Convent in Lisbon, with its rich historical significance and architectural beauty, epitomises the challenges faced in conserving such structures. The convent, a poignant symbol of Lisbon’s past, stands as a testament to the city’s resilience and heritage. This is especially important given its state of ruin after the 1755 Lisbon earthquake. However, its structural integrity and safety pose significant concerns, particularly in an urban area with a high population density and frequent tourist visits.

Traditional destructive testing methods are unsuitable for assessing the structure due to the potential for irreversible damage. Therefore, this study employs non-destructive testing (NDT) and structural health monitoring (SHM) techniques, alongside advanced numerical modelling, to evaluate the condition of the convent and ensure its preservation.

A significant aspect of this research is the focus on induced vibration monitoring and dynamic identification tests. The presence of a metro line underneath the convent introduces additional challenges, as vibrations induced by metro activities could potentially compromise the structure’s stability in sight of the weak soil condition, as was demonstrated by geological evidence and by the architectural changes that occurred in the structure [1,2,3,4,5]. Thus, the implementation of metro vibration monitoring (MVM) is crucial to assess the impact of these vibrations and to ensure they remain within safe and low limits based on foreign standards. The scarcity of specific standards for historical structures further complicates the engineering assessment required in this study, which needs to be supported by numerical and quantitative evidence. Hence, the research also incorporates a combination of finite element method (FEM) and discrete element method (DEM) numerical modelling to gain deeper insights into the current state of the convent. This comprehensive approach aids in the structural preservation of the church ruins of the Carmo Convent but also provides valuable data to inform stakeholders in decision making regarding protective measures.

**Figure 1:**
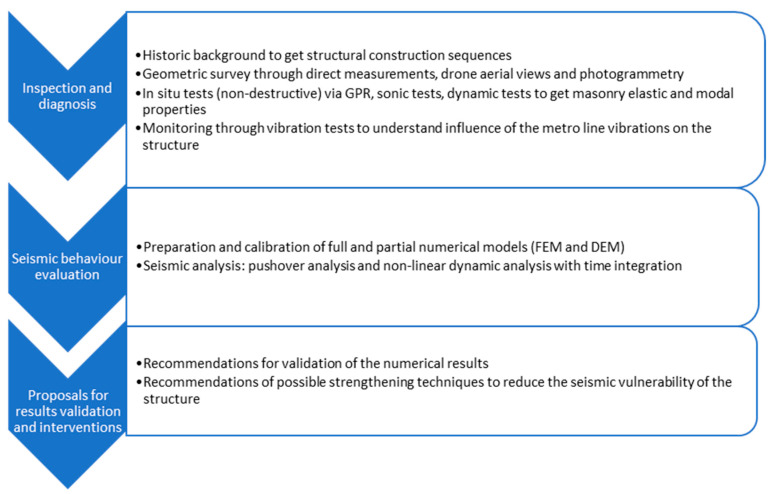
Flowchart of methodology undertaken.

For the sake of clarity, a methodology flowchart is presented in Figure 1. In the conservation process of Carmo Convent heritage, several steps have been undertaken to preserve its integrity. The inspection and diagnosis phases identified the main causes of damage and vulnerabilities from various perspectives, such as geometry, materials, and structural aspects. For the sake of heritage purposes, those steps were non-destructive. Following this, the structural capacity for the seismic action was evaluated. This methodology is designed to ensure informed decision making for interventions, aiming to minimise any destructive actions on the structure. This paper is mainly focused on the first two phases. However, it also presents recommendations for the validation of the results obtained from the numerical modelling and possible strengthening techniques to reduce the seismic vulnerability of the structure.

The content of this research is presented as follows: Section 1 is the present introduction to the scope of the study, while Section 2 introduces the object of study. Section 3 and Section 4 focus on the NDT and induced vibration monitoring, stressing the pros and cons given by the current state of the Carmo Convent. Once the most dangerous risks that can compromise the structural stability of the convent were identified, in line with the NDT testing results, two numerical models, namely a global finite element model (FEM) and a partial discrete element model, were built and calibrated using a methodology that better represents the vertical loading distribution, the lack of transversal elements and the absence of a roofing system. This is presented in Section 5. These aspects help to comprehend a very complex structure that has endured numerous seismic events over its lifetime, since the Carmo Convent suffered severe damage during the 1755 Lisbon earthquake and has been only partially reconstructed, becoming a symbol of that historic event.

## 2. Church Ruins of Carmo Convent

The church ruins of the Carmo Convent (national monument) are located in Lisbon, the capital city of Portugal, on the Iberian Peninsula. The elevation of the city ranges from sea level up to 102 m, but the Carmo Convent itself is at an elevation of about 42 m [6].

D. Nuno Álvares Pereira, the second constable of the kingdom of Portugal, funded the first construction works of the Carmo Convent, which began in 1389. However, the first and second attempts of construction were doomed to failure. The sandy soil on the site and its location atop one of Lisbon’s numerous hills prevented laying a successful foundation [7]. Master builders, commissioned to tackle the issue, found a solution in stepping the foundation to prevent the shear failure of the soil layers. The church was finally completed in 1397, but not without modification of the original plans. During construction works, a large crack appeared at the south side of the main chapel, and thus, flying buttresses were added to increase lateral support [8].

**Figure 2:**
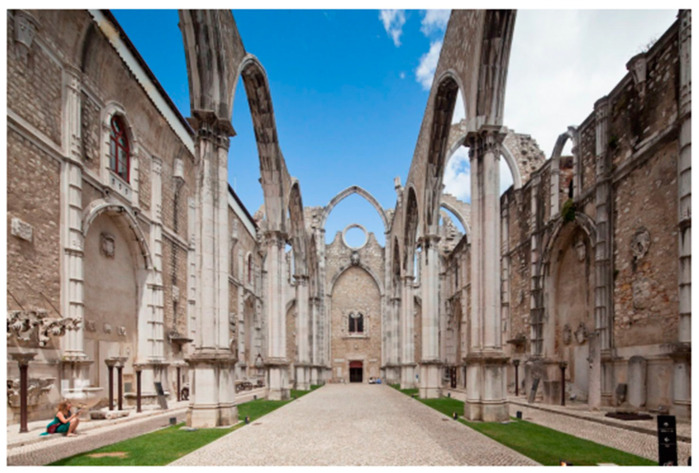
Church ruins of the Carmo Convent, Lisbon.

**Figure 3:**
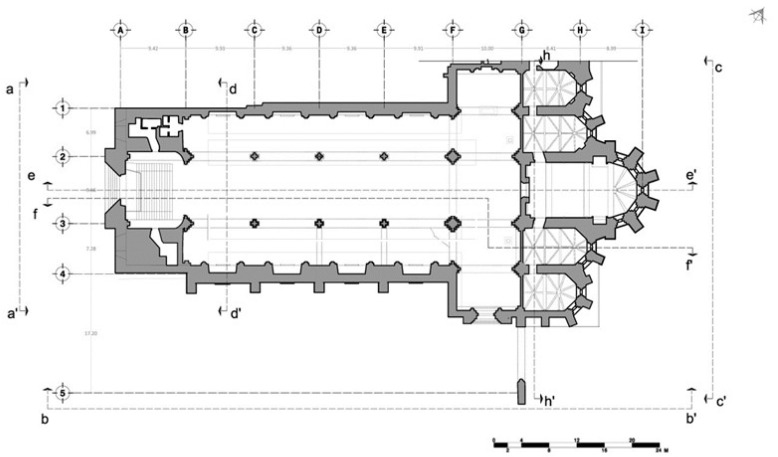
Floor plan of the church of the Carmo Convent.

**Figure 4:**
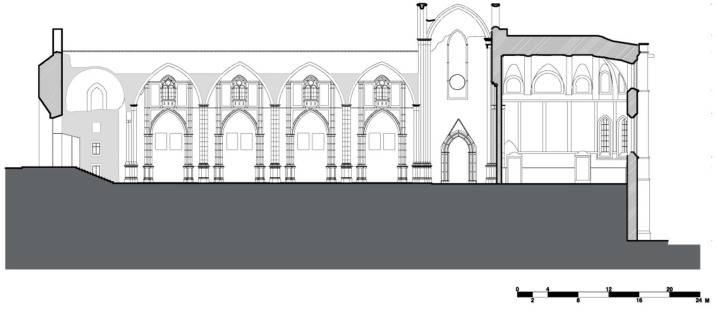
Section view of the interior (ee’) of the church’s nave featuring the arcades.

**Figure 5:**
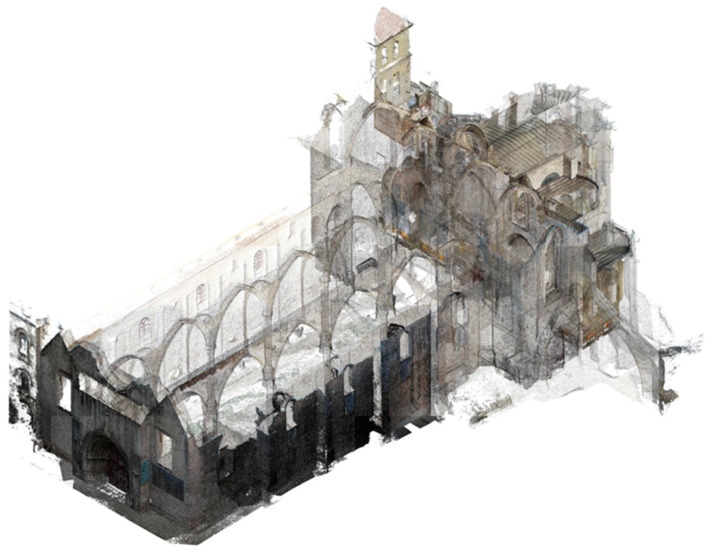
Perspective view for the church of the Carmo Convent (point cloud).

**Figure 6:**
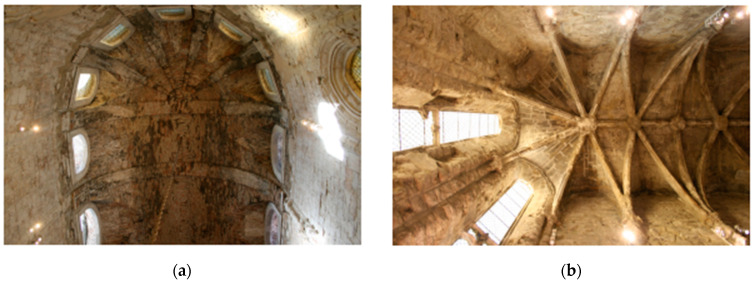
Details of the chapels: (**a**) masonry barrel vault covering the main apse; (**b**) example of the limestone ribbed vaults covering the secondary chapels.

The limestone masonry church of the Carmo Convent is made in the typical medieval style. The layout is a Latin cross plan (Figure 2, Figure 3, Figure 4 and Figure 5) with three naves making up the longitudinal section capped by five staggered semicircular apse and apsidal chapels and transepts on either side of the naves, and the church’s sacristy, which now serves as the museum store, protrudes off the northern transept [7]. Presently, the church’s apse has been transformed into an enclosed museum exhibition space that is covered by masonry vaults. A ceramic brick barrel vault with an apex approximately 17 m above the floor encloses the apse (Figure 6a), while the lesser chapels beside it are covered by limestone cross-rib vaults at heights of 13.40 m and 9.0 m stepping out from the central chapel (Figure 6b). It is unknown if the heights of these vaults are true to the original construction or if they were altered during restoration works. It is important to note that the northernmost chapel on the rear façade protrudes into the adjacent building, as does the clerestory [9].

On the church’s interior, the naves are divided by eight poly-style columns with 8.10 m spacing (longitudinal direction) supported by two rows of reconstructed arches and spandrel infills 15.6 m in height, which are 8.44 m (transversal direction) apart from each other (Figure 3 and Figure 4). The columns are approximately 1 m in diameter, except for those that support the largest arch, which are approximately 2.2 m in diameter. The transversal arch that connects the two rows and acts as an entrance to the transept is 23 m tall from the intrados of its keystone (Figure 2) [9].

The Carmo Convent became a ruin following two major seismic events that hit the structure in 1531 and 1755 A.D.

The written record gives special importance to the 1531 earthquake (~7.1 Mw), as Lisbon was a critical international port of commerce at that time. Letters, poems and official documents describe the devastating effects of the seismic shocks and the subsequent tsunami. The earthquake struck on January 26 between 4 and 5 A.M., killing approximately 1000 people in Lisbon and causing extensive damage to the city’s buildings. Approximately 1500 homes were lost, and severe damage was observed on many significant public buildings, such as the Ribeira Palace and Jerónimos Monastery [10]. The church of the Carmo Convent suffered damage as well, resulting in reconstruction works and the additions of a refectory and second cloister [7]. A library, refectory and second cloister were among these additions, and by 1551, the convent was repaired.

The earthquake that struck Lisbon on 1 November 1755, is one of the most destructive to have occurred in Europe, and it was followed by a tsunami and then a fire that further destroyed the church. At the Carmo Convent, the entire Library, consisting of 5000 volumes, was lost; the roof of the main church, the transepts and part of the nave collapsed; and the 126 monks and priests residing there were forced to relocate. The church was partially reconstructed but never wholly rebuilt as anti-clericalism gained ground in the latter part of the 18th century. Instead, the church was transformed into a museum [7]. During this period, the ribs, arches and pillars were added in the neo-Gothic style as they presently exist.

Among the recent interventions through the centuries, the ones that took place in the 1940s and 1950s were the most significant. The walls were restored, and five windows were opened within them, some with wooden and glass windows and others with stained glass. Concrete walls were removed and replaced with lime mortar and masonry. The missing columns were installed, and the church walls were consolidated. In 1954, the arch of the main nave was disassembled so that the walls could be straightened. In 1955, concrete was used on the façades and arches to consolidate the stonework.

In addition, in 1995, an underground railway was proposed to run beneath the convent. Studies on the silty foundation soil layers were conducted to assess the potential impact on the convent. Due to concerns about the condition of the foundations, numerical models and in situ testing revealed that the soil would be close to plastic failure if the tunnel were to be built. Consequently, 164 micropiles were installed to stabilise the foundations. However, this intervention negatively affected the upper uncompressed soils, which required correction with jet grout injections in 2004 [1]. These developments stressed the need to analyse the current condition of the structure’s dynamic response following these interventions.

## 3. Non-Destructive Tests

Non-destructive testing (NDT) methods allow for a detailed assessment of the current condition without causing further damage to the already delicate remains of the convent. In addition, the complex history of the Carmo Convent, including damage from the 1755 Lisbon earthquake and subsequent partial reconstructions, necessitates a thorough understanding of structural integrity at present. NDT techniques can reveal hidden flaws, such as internal cracks or voids, which could compromise the stability of the convent. NDT provides precise data on how the convent might react to such forces, enabling engineers to design appropriate reinforcement measures. This proactive approach helps preserve the historical and cultural significance of the Carmo Convent while ensuring its long-term stability and safety.

For the diagnosis of the Carmo Convent, multiple types of NDT and investigations were carried out to understand the materials, physical properties and vibrations. The non-destructive tests performed on the Carmo Convent included sonic testing, vibration testing, dynamic identification tests and ground-penetrating radar (GPR) scans.

### 3.1. Geometrical and Damage Survey

A comprehensive geometrical and damage survey of the Carmo Convent was carried out by means of orthographic photos and drone aerial photos.

The geometrical survey was created by a 3D point cloud modelled in Photoscan [11] with the aid of orthographic photos surrounding the work field from outside to inside and from the bottom to the top. The minimum overlap adopted was 60% for each shot. The photos were taken with a digital camera with the properties in manual mode and the focus in automatic mode. The specifications of the photos are an exposition time of 1/500 s, with an ISO of 100 and an aperture lens of 3.6 mm. Photos were also taken with a DJI drone belonging to the University of Minho. The drone was used to obtain clear photos of the higher elements of the convent, which could not be accessed due to site restrictions.

The drone photos were also taken with the same settings to ensure that all photos could be merged. In some cases, the photos presented differences in ISO or exposition time/aperture of the lens during the photogrammetry process in Photoscan. The Optimization camera command was used to match the properties and calibrate the direction of the photos. Furthermore, the photos were taken in each room or open space (chapels, library, naves, apse, exterior façades, etc.).

The photos were optimised and merged for the creation of a dense point cloud, detected automatically with global coordinates, namely the coordinates that belonged to the drone shot. Due to this aspect, the coordinates of some photos were transformed from local to global coordinates.

The percentage of error obtained by the model was between 0.5% to 0.6%. A mesh was generated and texturised in order to obtain orthographic views for façades and sections used in the damage survey. Nevertheless, to process the model in three dimensions, Rhinoceros 5 software [12] was required, and adjustments of scale were performed. From Rhinoceros, the principal points of the model were exported in a text file and imported in an AutoCAD file, aiming at improving the references and minimising the error rate. Next, the drawings were rectified and scaled with the proper proportions. From the dense cloud point, a mesh was created in Photoscan, and then the texture was applied. Subsequently, the cloud point was exported as a “.las” extension in order to import those points in ReCap 360 software [13] to take the measurements with the created points. This model was exported to an AutoCAD file to obtain an accurate geometry of the Carmo Church (see Figure 3). From the 3D geometric models, the sections and elevations were generated in AutoCAD. These results were used for several tasks in this work, such as damage patterns and numerical modelling (Section 5).

**Figure 7:**
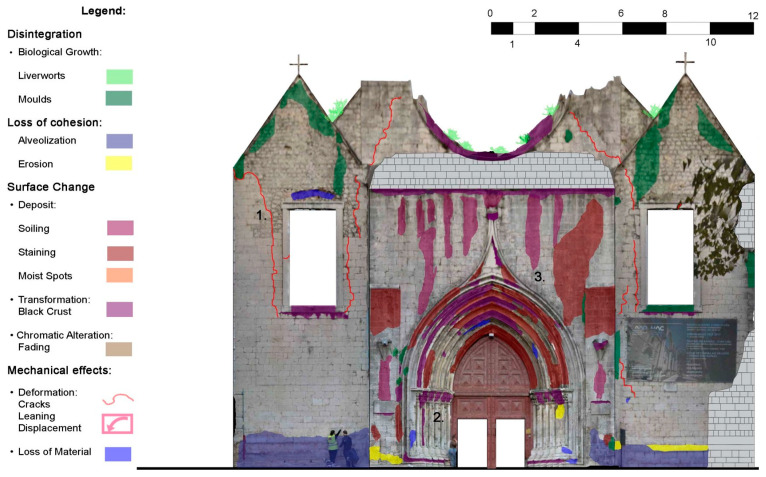
Example of damage pattern for the main façade before conservation and restoration works.

The damage surveys were performed by means of the aerial photogrammetry survey, which produced the images of the façades and interior walls used in the damage surveys (main façade in Figure 7). In-depth visual inspection surveys for each of the chapel’s four elevations, both for the interior and exterior surfaces, were performed. The results were plotted onto the photogrammetry images. The damage survey assesses the extent of the damage and/or loss observed during the visual inspections. It is noted that Carmo Church presented damage caused by several seismic events, which was repaired after the 1755 earthquake. The damage is mainly associated with material decay over time. The damage survey allowed us to prepare the damage patterns based on the ‘Illustrated Glossary on Stone Deterioration Patterns’ [14]. The church of the Carmo Convent does not show severe damage, but some aspects have to be taken into account. The south-east corner is the most affected by decay, and some problems are related to the architectural changes that occurred during the history of the Carmo Convent. Moreover, some damage is associated with the properties of soil and the partial slope failure of Carmo hill induced by the 1755 Lisbon earthquake [1]. The main effect is visible on the portals, which are heavily broken down and associated with a global leaning. It is noted that, after this work, conservation and restoration works were performed in the main façade and Gothic arch.

### 3.2. Sonic Tests

Sonic testing was performed throughout the convent to estimate the material properties of the stonework. Direct and indirect sonic testing was used to estimate Young’s moduli of the walls, columns, buttresses and façades. Sonic testing involves measuring the velocity of generated waves through a wall or column. These waves are generated using an impulsive hammer and measured using an accelerometer. Two types of tests were carried out, namely direct and indirect tests. Direct sonic testing was performed where both sides of the wall or column were easily accessible, while for indirect sonic testing, the hammer and accelerometer were placed on the same side of the element being examined. Indirect testing provides the velocity through the surface of the element. For direct sonic testing, the primary waves are used to determine the velocity, signified as *V_P_*, while for indirect sonic testing, the Rayleigh waves are used, with the velocity signified as *V_R_* [15]. From the obtained velocities, Young’s modulus *E* can be obtained. The Poisson’s ratio *v* was assumed to be 0.2, while the density was obtained by averaging the values proposed by the Italian code [16], which resulted in a density *p* of 2039 kg/m^3^.

From these values, Young’s modulus is calculated through the following Equations (1) and (2) [17]:

(1) $$E=\frac{{\rho \cdot V}_{p}^{2}\cdot (1+v)\cdot (1-2v)}{\left(1-v\right)}\;\;(\mathrm{direct}\mathrm{tests})$$

(2) $$E=\frac{\rho \cdot V_{R}^{2}\cdot 2(1+v)\cdot {\left(1+v\right)}^{2}}{{\left(0.87+1.12v\right)}^{2}}\;\;(\mathrm{indirect}\mathrm{testing})$$

**Figure 8:**
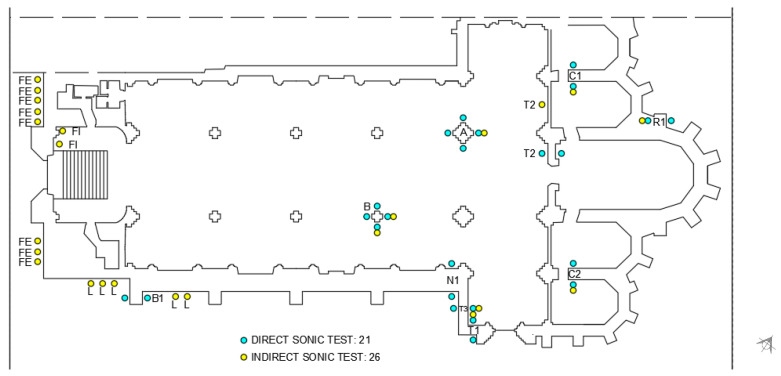
Sonic test locations.

The elements that were measured using sonic testing included two columns, seven walls and two buttresses. A total of 21 direct and 26 indirect tests were performed. These tests were performed multiple times at each of the positions, with the average velocity and Young’s moduli obtained for each. The location of each of the tests is presented in Figure 8, and Table 1 shows the results obtained from the direct and indirect sonic testing.

The results included very high direct sonic test results in comparison to the indirect ones. This may be related to the uncertainties in the determination of the arrival of the R waves for the indirect sonic tests and location with a high percentage of solid units (stones) in comparison with the mortar in the direct sonic tests. Besides that, these results are used as initial values for the numerical modelling because the results obtained from the indirect tests are closer to the expected values found in the literature. Afterward, the material properties are calibrated based on the modal properties estimated from the dynamic identification tests.

### 3.3. Dynamic Identification Tests

Determining the natural frequencies, mode shapes and damping ratios of the structure allowed for further calibration of the numerical model in an effort to create a realistic model that gives insight into the possible seismic performance of the church ruins of the Carmo Convent and its possible deficiencies and failure mechanisms. The data collection was performed using the output-only method relying on ambient ground vibrations to excite the structure, which was monitored with piezoelectric accelerometers, coaxial cables and data acquisition system software developed by the University of Minho [18]. The accelerometers that were used have a sensitivity of 10 V/g, a frequency range of 0.15 to 1000 Hz and a dynamic range of ±0.5 g. The data were collected in single (the arcades and columns) and multiple acquisitions (chapels and lateral wall) that lasted 20–30 min due to the large dimensions of the building and the on-site available means of access for these tests.

The dynamic identification tests aimed to determine the dynamic properties of the most vulnerable parts of the church (e.g., the freestanding arcades and columns), also compared with the stiffest portions of the convent (e.g., chapels and lateral wall).

**Figure 9:**
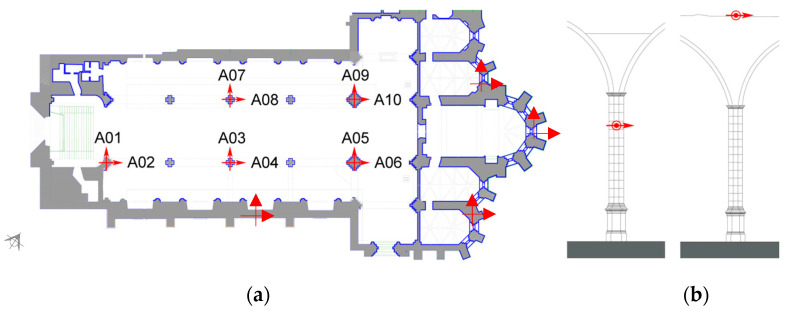
Indicative testing setup of the dynamic identification tests: (**a**) plan; (**b**) height for accelerometers on the north arcade and the south arcade (arrows indicate reference axis of the accelerometers).

The freestanding nature of the arcades and columns not only makes them structurally vulnerable, but their access is also difficult for dynamic identification testing. However, the arcades are slender and mostly unaffected by the main structure. To access the tops of the arcades, it was necessary to hire a professional climber to place the accelerometers safely. For this reason, the installation of the accelerometers on the south arcade took a considerable amount of time, so the accelerometers on the north arcade were installed with a ladder on the column faces rather than the top of the arcades. The testing plan utilised 10 accelerometers placed in pairs to measure the response in both the transverse and longitudinal directions at the arcades (Figure 9).

To estimate the dynamic properties of the church of the Carmo Convent, the enhanced frequency domain decomposition (EFDD) method was used. The EFDD method corresponds to a method with decomposition in the frequency domain and is divided into two main steps. First, the EFDD peak picking is performed. Then, the EFDD-identified mode shapes are used to identify the SDOF spectral bell functions. From these SDOF spectral bells, both the frequency and damping ratio are estimated.

**Figure 10:**
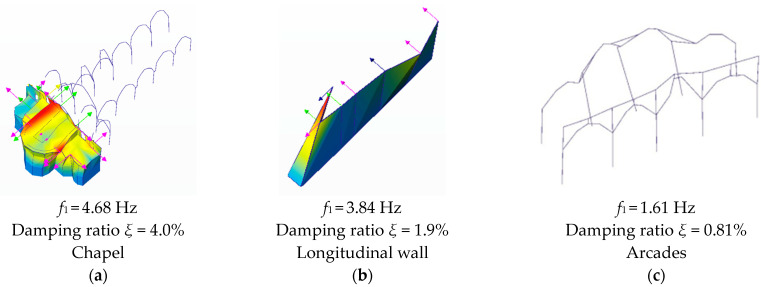
First experimental modal shapes estimated from the dynamic identification tests: (**a**) for the chapels; (**b**) for the southern longitudinal wall; and (**c**) for the arcades (arrows corresponds to the accelerometer locations).

Regarding the chapels, four modes with a frequency ranging from 4.68 Hz to 12.24 Hz were estimated. The first mode (4.68 Hz) corresponds to the first global mode of the chapel in the transversal direction of the church (Figure 10a). The second mode (6.59 Hz) is the second global mode of the chapel in the longitudinal direction of the church. Mode 3 (8.60 Hz) is also a global mode, corresponding to a distortional mode of the chapel in the transversal direction of the church. The last mode (4th mode–12.24 Hz) is the first local mode of the central part of the chapels in the transversal direction of the church.

Regarding the longitudinal wall, a further four modes with a frequency ranging from 3.84 Hz to 7.24 Hz were estimated. The first mode (3.84 Hz) corresponds to the first local mode in the longitudinal direction of the church (Figure 10b), which can be associated with the local mode of the main façade. The second mode (4.35 Hz) is the first local mode of the longitudinal wall in the transversal direction of the church, with the highest amplitude close to the transept. Mode 3 (5.71 Hz) is also a local mode, corresponding to a transversal mode with a single curvature. It is noted that this wall was completely reconstructed after the 1755 earthquake. The last mode, Mode 4 (7.24 Hz), is the local mode of the wall in the transversal direction of the church, with a second curvature.

Lastly, regarding the arcades, five modes were estimated. Local modes of 1.61 Hz and 3.05 Hz correspond to transverse and longitudinal modes, respectively, on the north arcade (Figure 10c). Local modes on the south arcade were found to be 2.26 Hz in the transverse direction, 3.57 Hz for the first curvature and 3.91 Hz in the longitudinal direction. These frequencies were considered to be within the acceptable range of the structure. Peaks above the frequency of 3.91 Hz were considered too high to be accurate, as higher resonant frequencies are stiffer and thus more difficult to excite through ambient vibration alone. Furthermore, the first modes have the highest contribution to the dynamic behaviour of building structures [19].

Figure 10 shows the first modes of the three tested locations.

### 3.4. GPR Surveys

**Figure 11:**
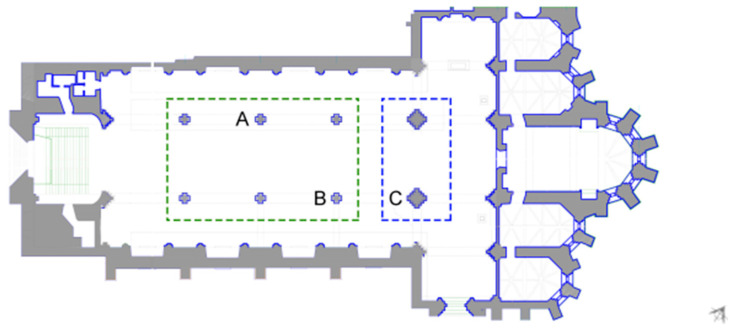
Floor plan of the church of the Carmo Convent depicting the columns chosen for GPR testing (A, B and C).

Ground-penetrating radar (GPR) was used to garner a better understanding of the arcade columns that divide the church’s three naves. GPR was performed on three of the arcade columns. The columns within the green box in Figure 11 are essentially identical; thus, only two were chosen for GPR, namely columns A and B. In the blue box, the two larger columns are also assumed to be identical, so only the column labelled C was analysed with GPR.

**Figure 12:**
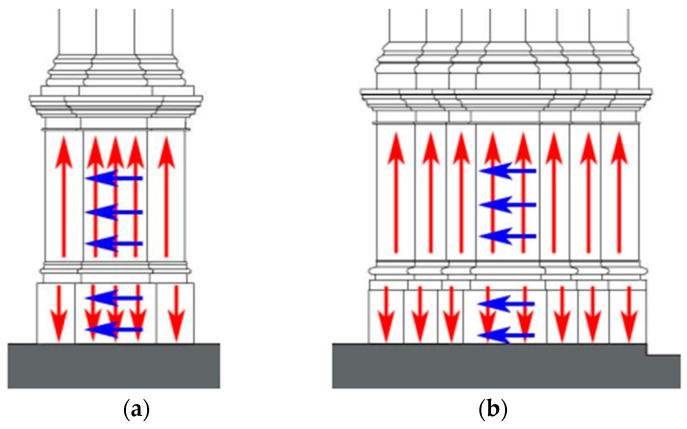
Drawings of the bases of the two column types: (**a**) columns A and B in Figure 11 and (**b**) column C in Figure 11. The red arrows show the vertical scans taken on the columns, and the blue arrows show the horizontal scans.

The goal of the analysis was to understand the dimensions and potential interlocking of the limestone units used for the base of the columns. Thus, the scanning process shown in Figure 12 was adopted, where the red arrows represent vertical scans and the blue arrows represent horizontal scans. Here, the direction of the arrows indicates the direction in which the antenna was oriented. Using a 1.6 GHz antenna that can record depths up to 1 m, ten scans were taken in the vertical direction and five scans in the horizontal direction on each of the four faces for columns A and B. For column C, 16 and 6 scans were made on all four faces vertically and horizontally, respectively. The redundancy of these scans protects against misinterpretations or missing data due to bad or noisy readings. In addition, scans taken orthogonally allow for evaluation of the stone arrangement and the inaccessible interiors of the columns.

**Figure 13:**
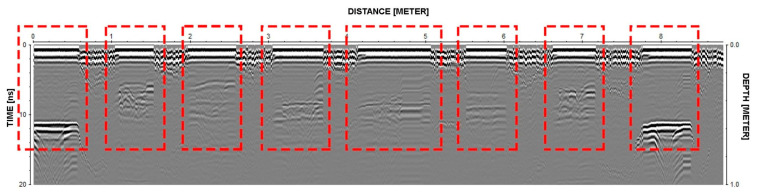
Data acquired by a scan on column C, representative of the vertical scans taken on the columns. The data shown in red dashed rectangles correspond to the red arrows shown in Figure 12b.

The clarity of the scans (Figure 13) made it reasonable to conclude that the stones seen on the column’s exterior are solid and extend approximately 28–30 cm into the column. Beyond the singular stones, the scans are less clear. These portions of the scans, represented by the hatch, show significant wave reflection, which indicates that the interiors of the columns are likely not made of solid stone, nor does it appear that these are clean-cut stone blocks fitted together. Rather, it is likely that the interiors are at least partly filled with limestone rubble and compacted with good-quality mortar. It is possible that the upper portion of the column extends into the base or that there are larger solid stones at the interior that were not reached by the electromagnetic wave from the GPR.

Due to accessibility limitations, the upper parts of the columns were not scanned using GPR. However, educated assumptions can be made based on the results of the bases and similar structures. Most likely, large stones make up most of the cross-section, with just a small area at the core that is rubble. This high percentage of limestone in the columns’ cross-section and the thin mortar layers would allow for the high modulus found by direct sonic testing [9], also confirmed by the calibration of the numerical model (Section 5). These assumptions were also confirmed by additional works carried out on the arcades by Secanellas et al. [20]. Secanellas et al. [20] used a novel ultrasonic tomography system (UTS) along the height of Carmo Convent columns. The results obtained showed valuable information on internal cracks and the overall degradation state of the inspected drums of the columns. Moreover, the results also allowed an understanding of how the columns were precisely constructed, showing that the shaft is constituted by single blocks of limestone.

## 4. Vibration Assessment

The primary source of vibrations affecting the church ruins of the Carmo Convent comes from the moving metro trains directly under the site. This must be investigated, taking into account the details of the unevenness of wheels and track lines. Moreover, irregularities could cause impact excitation in rail joints and wheel flats [21]. This kind of vibration creates signals that reach a nearby structure and is defined as ground-borne vibration, showing, usually, ranges from 30 to 250 Hz, with a particle velocity range of 0.2–50 mm/s considered sufficient to cause structural and aesthetic damage [22]. Despite the consolidation works, and given the slenderness of the ruins of the Carmo Convent, the effect of induced vibrations still remains a source of concern for the conservation of the structure. Hence, in order to verify the potential cause of damage, measurements of induced vibrations during metro operation hours were carried out.

Current international standards cover many aspects of the assessment for induced ground-borne vibrations, including proper instrumentation, recording, signal processing and evaluation and threshold limits [22]. In specific, threshold limits of kinetic quantities are defined in terms of the type of structure, often with a specific category for historical buildings or buildings sensitive to vibrations, and according to the dominant frequency of the ground-borne vibration input. The main goal of a ground-borne vibration assessment consists of comparing the peak particle velocity at the point of concern, either the maximum in any direction *V_max_* or the maximum of the vectorial sum *V_sum_* given by Equation (3) [1], with the threshold levels from the adopted standard. As described in [19], the process of assessment of ground-borne vibrations in structures is based on the direct measurement of particle velocities under high sampling frequencies of around 1000 Hz, compared to threshold values from international standards. Additional acquisitions without metro operation can also provide reference values on particle velocities and the range of frequency content to be used for band-pass filtering [23].

(3) $$V_{sum}=\sqrt{V_{xi}^{2}+V_{yi}^{2}+V_{zi}^{2}}$$

**Figure 14:**
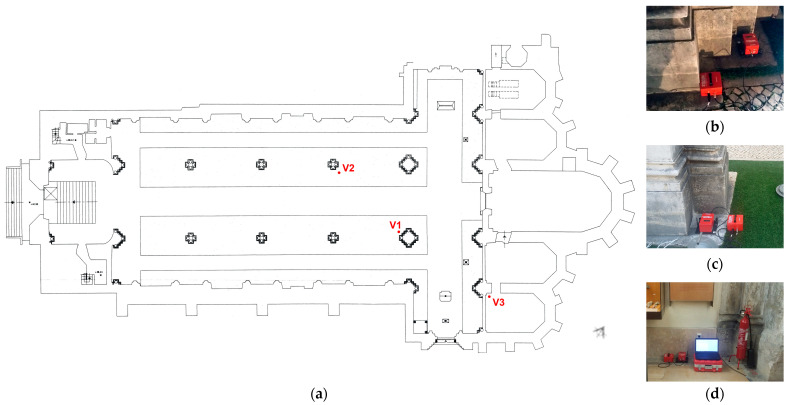
Plan view of church of the Carmo Convent: (**a**) with testing locations of acquisitions of induced vibrations V1 (**b**), V2 (**c**) and V3 (**d**).

The ground-borne vibration tests were carried out within a time window of 3 days, placing a vibration measurement system, a triaxial velocimeter-geophone (MR3000C), at the bottom of two selected piers (V1 and V2 in Figure 14) and inside the south chapel (V3 in Figure 14). The geophone has a recording sampling range of 250–4000 Hz, a measurement velocity range of ±100 mm/s and a frequency range of 1–350 Hz. Vibration recordings were taken from 6:00 pm to 01:00 am in the first measurement (V1), from 7:00 pm to 09:00 am in the second one (V2) and from 11:00 am to 01:00 pm in the third one (V3), with time durations setups of half hour and a sampling rate of 1000 Hz.

**Figure 15:**
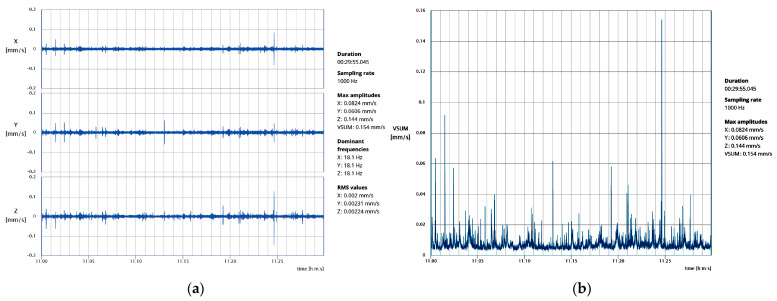
V3 acquisition during 11:00–11:30 am: (**a**) time plot of particle velocities in three directions; (**b**) time plot of vectorial sum.

**Figure 16:**
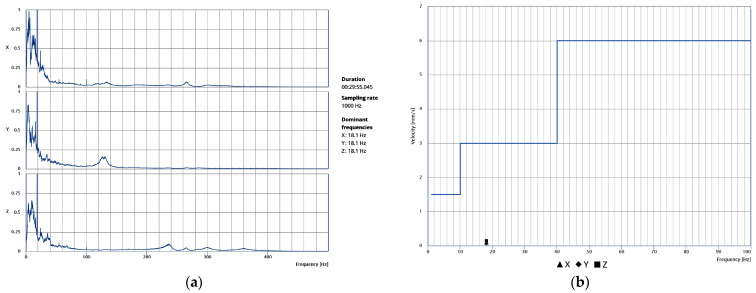
V3 acquisition during 11:00–11:30 am: (**a**) FFT plot; (**b**) maximum particle velocities and limit thresholds from NP 2074 [23].

For V2, the acquisitions in which the metro is not working (01:00–06:30 am) constitute a base reference for comparison during the post-processing. Here, basic statistics for absolute particle velocities in each direction with no metro operation are presented in Table 2. Considering the median values, the base zero reference particle velocities are in the range of 7–11 μm/s. Further, Table 3, Table 4 and Table 5 present basic statistics of absolute particle velocities during metro operation for V1 (6:00 pm to 01:00 am), V2 (7:00 pm to 01:00 am and 06:30 am to 09:00 am) and V3 (11:00 am to 01:00 pm), respectively. The maximum recorded particle velocity is found in V3, equal to 0.1441 mm/s, with the corresponding time-history plots in all directions and the vectorial sum presented in Figure 15a,b, respectively. For the specific signal, the fast Fourier transform (FFT) plot in Figure 16a reveals the dominant frequency, with the highest energy content equal to 18.1 Hz. Thus, accounting for NP 2074 [24], with a maximum particle velocity limit for sensitive structures and a dominant frequency range of 10–40 Hz, is equal to 3 mm/s. Thus, the maximum recorded particle velocity is found to be 21 times lower, as also presented in Figure 16b. In conclusion, the structure is not affected by the metro operation for the monitored period, also accounting for the low speed of trains moving, as the metro station is located directly below the structure.

## 5. Seismic Vulnerability

Although the dynamic effects associated with the underlying metro are not significant, among the Iberian Peninsula, the Lisbon area is the most prone to earthquakes [25,26,27,28,29,30] due to its proximity to the boundary of the Eurasian and African plates. Thus, the study of seismic vulnerability is crucial for the church ruins of the Carmo Convent. Indeed, a study performed by [25] produced maximum intensity maps for peak ground acceleration in Portugal, which shows that Lisbon has the potential for earthquakes up to intensity X on the EMS-98 scale [31]. Because of that, several studies have been performed on the structural behaviour and significance of the Carmo Convent.

In 1995, Gil and Oliveira [6] performed a study to explain the likely behaviour of the structure during the infamous 1755 Lisbon earthquake. A linear numerical analysis based on FEM was performed. The model was built to represent the structure as it was prior to the 1755 event. Iconographic sources, a technical model by the municipality and in situ measurements of geometry and ground vibrations were used in this work. The ground vibrations were intended to identify anomalies in the geotechnical foundation, but none were detected.

Lemos (1997) [32] also utilised numerical modelling methods to analyse the convent. This study used the discrete element method (DEM) to focus on the behaviour of the arcades’ masonry arches. The arches were modelled independently without the interaction of the filling or columns. The work also neglected the tensile strength and cohesion in joints by assuming they are elastic. Self-weight and in- and out-of-plane dynamic loads were applied to evaluate the load capacity of the partially confined semicircular and pointed arches. The results of the dynamic analyses depict a capacity for the pointed arches varying from 0.47 to 0.61 g for the in-plane response and from 0.47 to 0.67 g for the out-of-plane response. These values are, on average, 50% higher than the ones obtained through static analyses.

Here, two different numerical modelling approaches, a global macro-FEM model and a partial DEM model, focused only on the most seismically vulnerable part of the structure, the north arcade, are used to evaluate the seismic response. Both models were calibrated based on the experimental results.

### 5.1. Finite Element Model

**Figure 17:**
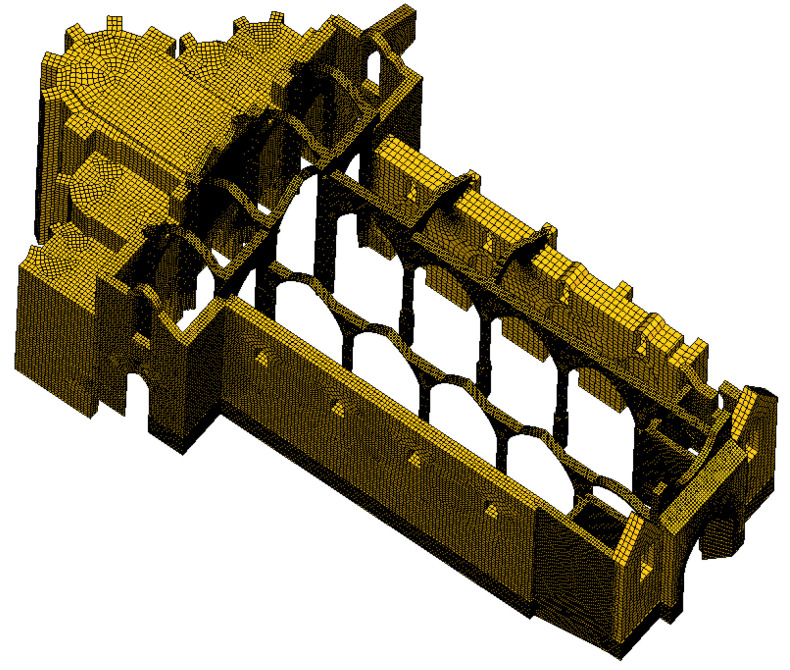
FEM global model in DIANA environment.

A global finite element model of the church ruins of the Carmo Convent was created in DIANA 10.8 software [33] based on the geometry obtained through the aerial photogrammetry model. In terms of connections, full connectivity between parts was assumed (Figure 17).

As is the case with any complex structures, several simplifications with respect to the geometry obtained from the point cloud and assumptions were made, taking into account the adopted modelling strategy and the objectives of this study. In particular, the foundation was assumed to be 3 m deep with no enlargement from the observable floor plan; the adjacent building was not accessible for a geometrical survey and was not considered in the model (conservative assumption) even though it is likely to affect the behaviour of the structure, namely the overturning collapse of the north longitudinal wall; the geometry of the north longitudinal wall was approximated, assuming an approximate symmetry with the south wall; the geometry of the cross-sections of the arcade columns was simplified to improve the performance/stability of the model/mesh; the cross-sectional area of the simplified version was kept equal to the original ones of the actual columns; and the vaults in the chapels were simplified into barrel vaults rather than complex cross vaults, which cause difficulties in the FEM mesh generation, considering that they are less seismically vulnerable, that this simplification has little impact on the results and that the chapels were considered symmetric along the east–west axis.

The final mesh was created using an automated method. Initially, the mesh was generated for the entire geometry with an element size of 0.2 m. However, this resulted in over 1,500,000 elements, which was unmanageable. To address this, the element size was adjusted to 0.4 m for the walls while keeping it at 0.2 m for the pillars, arches and filling. This modification reduced the total number of elements to approximately 500,000. Considering the model’s complexity, although solid quadratic elements (CHX60) could be used, a solid linear interpolation element (HX24L) was chosen. Boundary conditions were applied to the base of the walls, assuming a fixed connection to the soil.

The material properties that are necessary for the model include Young’s modulus, density, Poisson’s ratio, compressive and tensile strengths and compressive and tensile fracture energies. The FEM model presents six different materials, which correspond to the arcade columns; arcade arches; arcade filling; poor filling in the chapels; longitudinal walls; and transverse walls, nave room walls and façades.

The properties that were not calibrated were estimated according to indirect sonic testing, the Italian building code (NTC 2008) [16] and an evaluation of the masonry quality index (MQI) [34].

The masonry quality index was also used to determine the material properties of the masonry. This index is a subjective analysis that provides values for compressive and shear strength. The method involves visual inspections based on several factors, including the stone’s mechanical properties and conservation state (SM), stone dimensions (SD), stone shape (SS), wall leaf connections (WC), horizontal bed joints (HJ), vertical joints (VJ) and mortar properties (MM). For vertical loads, the analysis considered the columns, façade wall, longitudinal wall and chapels. The compressive strength was approximately 5 MPa for the columns and façade wall, around 1.3 MPa for the longitudinal wall and about 3.6 MPa for the chapel walls. In terms of shear strength, the columns and façade wall were approximately 0.7 MPa, the longitudinal wall was about 0.02 MPa, and the chapel walls were around 0.05 MPa.

The initial properties used for the structure before calculation were chosen to obtain a value of the first numerical frequency, which was reasonably close to the first experimental frequency. For the limestone masonry in the arcade columns and arches, given that they are made of good-quality masonry with thin mortar joints, the choice of Young’s modulus that approaches that of the limestone units alone, equal to 35,000 MPa, was considered reasonable. Following this, a calibration based on experimental and numerical frequencies and mode shapes was carried out.

First, linear elastic analysis was conducted, aiming at evaluating the model’s reliability (mesh) and the response of the structure for the vertical loading. Then, an eigenvalue analysis was also performed to obtain the numerical modal properties (frequencies and mode shapes), which were compared with the experimental modal properties obtained from the dynamic identification tests, in the calibration of the numerical model. Model validation is fundamental since incorrect assumptions and/or inaccurate results obtained in the inspection and non-destructive testing can lead to wrong conclusions on the seismic vulnerability of the structure. Thus, and taking into account the methodology used in the dynamic identification tests, three independent calibrations were performed for different parts of the building (south wall, chapels and arcades). In the calibration of the south wall, the foundation level (total height of the wall) and Young’s modulus of the masonry were adopted as variables to calibrate. On the other hand, in the other two calibrations, only Young’s moduli of the masonries were used as variables to calibrate.

For example, Table 6 presents the attempts carried out for the calibration of the south wall modal properties. After these attempts, the assumptions that better matched the experimental results were obtained for the foundation level of 3 m and Young’s modulus of the masonry equal to the one estimated from the sonic tests (16 GPa). This last high value was not adopted at the beginning of the calibration but after the calibration process seemed to be adequate (for the equivalent stiffness of the wall) and was adopted.

Once the experimental and numerical mode shapes were related, it was possible to perform an automatic calibration of Young’s moduli that minimises the difference between the experimental and numerical frequencies using the method proposed by Douglas and Reid (1982) [35]. A weighting factor to emphasise the calibration of the first mode was considered, mainly because the first mode has the greatest impact on the dynamic performance and can be identified experimentally with high accuracy.

Regarding the nonlinear material properties, the tensile strength was determined to be equal to 200 kPa. Again, this value was considered acceptable because the arches are made of very high-quality masonry. The tensile behaviour of masonry is assumed to be exponential, while the compressive behaviour is parabolic.

In the conventional application of the self-weight in a pushover analysis (application of the loading by steps using the full geometry), relevant stress concentrations arose at the arch connections, and the mid-span of the arches was observed (low tensile strength of the masonry and low axial force for the first steps). Thus, the use of the phased analysis, where it is possible to assign a sequence for the application of the different portions of the structure and their self-weights, was needed. Portions of the structure are assigned to a given phase (the arches were applied in the last phases), with the corresponding weight assigned in steps as a conventional static load application. Here, the number of utilised phases was chosen mainly to eradicate unrealistic damage in the structure due to the self-weight, besides as a better representation of the construction process. Thus, using the phased analysis, it was possible to significantly reduce the initial damage (maximum crack width) to the structure by 87%, from 1.3 mm to 0.17 mm, when compared to the corresponding conventional application of a uniform assigned self-weight for all structural elements. The material properties are summarised in Table 7.

**Figure 18:**
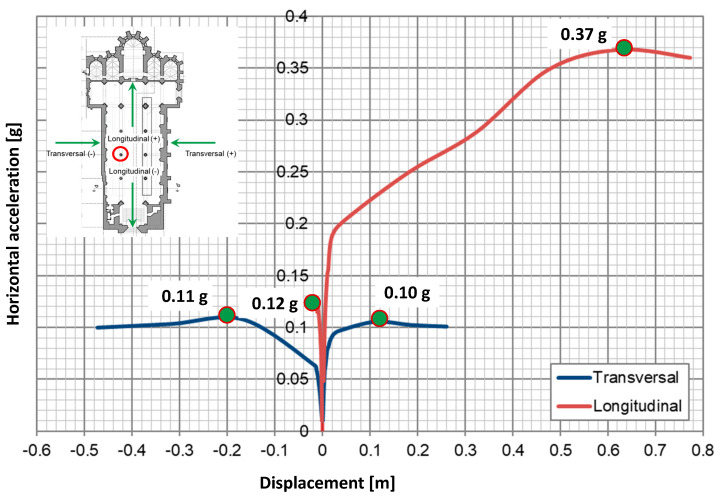
Capacity curves for the global FEM model (control point highlighted in red).

Using the phased analysis for the self-weight application, the pushover analysis for seismic action was performed on the entire FEM model (Figure 17) using a horizontal load pattern proportional to the mass. Given the complexity and asymmetry of the structure, pushover analyses were applied to the four directions: north, south, east and west. Henceforth, these directions will be discussed in terms of the relative outward direction in each elevation: north as the positive transverse direction, south as the negative transverse direction, east as the positive longitudinal direction and west as the negative longitudinal direction (Figure 18). The capacity curves presented in this study represent the applied horizontal acceleration (ratio between the sum of the horizontal forces proportional to the mass and the self-weight in “g”) as a function of the displacement at the control point, where the top of the highlighted column in Figure 18 meets the arcade fill. The location of the control point was based on the expected response of the structure, namely the most vulnerable elements and highest displacements.

**Figure 19:**
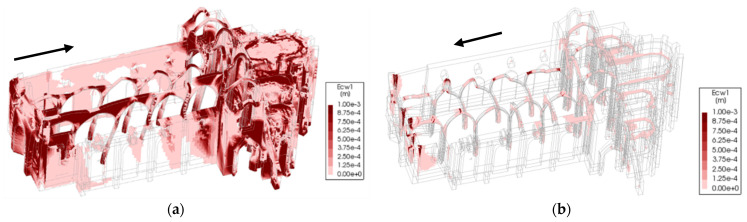
Pushover in the longitudinal direction for the FEM model (crack width resulting from pushover analysis: (**a**) positive longitudinal direction; (**b**) negative longitudinal direction (the arrows indicate the seismic action direction).

**Figure 20:**
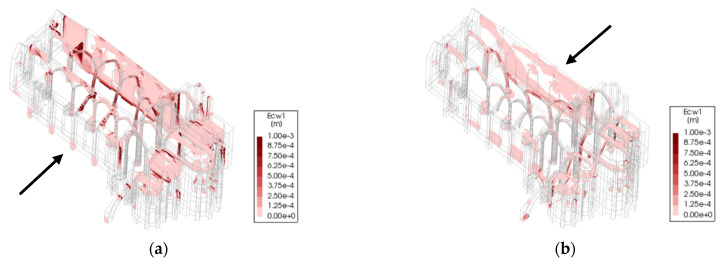
Pushover in the transverse direction for the FEM model (crack width resulting from pushover analysis: (**a**) positive transverse direction; (**b**) negative transverse direction (the arrows indicate the seismic action direction).

Accounting for the crack width plot in Figure 19 and Figure 20, the north arcade is the most vulnerable portion of the structure (0.10 g). The two transverse arches in the south aisle, connecting the south arcade with the lateral nave wall, are critical for providing transverse stiffness to the south arcade and improving its seismic performance.

Considering all the conducted pushover analyses, the least vulnerable for the structure is in the positive longitudinal direction (0.37 g), mainly due to the in-plane stiffness provided by the arcades, longitudinal wall and chapels. The arcades can deform more in their plane before the structure fails. However, severe damage is endured in the process (Figure 19a). Instead, in the negative longitudinal direction, the capacity is equal to 0.12 g, with the north arcade failing under comparatively little displacement due to the differences in stiffness and strength between the free span of the arches and the nave room walls together with the front façade (Figure 19b) given by the transverse arches. For the pushover applied in the negative transverse direction, the structure presents a low increase in capacity (0.11 g) with respect to the positive one (Figure 20b). Hence, the transverse direction structure is considered the most vulnerable for the structure, even if by a small amount (Figure 18 and Figure 20a). Hence, this direction was chosen as the subject of study for the DEM analysis.

**Figure 21:**
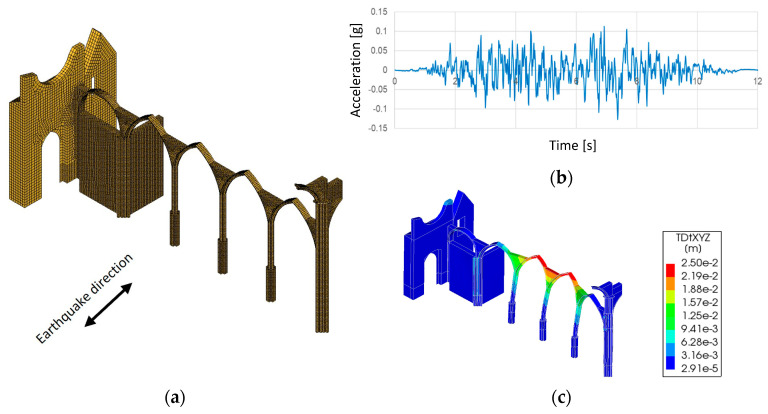
Nonlinear dynamic analysis: (**a**) partial FEM model of the north arcade; (**b**) accelerogram; (**c**) maximum displacements.

Finally, a nonlinear dynamic analysis with time integration was performed for a partial FEM model of the north arcade (Figure 21a). Taking into account the results obtained in the pushover analysis in the transversal directions, an artificial accelerogram, generated in the SIMQKE_GR 1.3 software and compatible with the response spectrum for Type 2 earthquakes of Eurocode 8 [36] and scaled for a PGA equal to 0.12 g (about the PGA expected for Lisbon and soil type C), was applied in the orthogonal direction to the arcade (Figure 21b). Rayleigh damping with coefficients equal to 0.522787 (α proportional to the mass matrix) and 0.000823 (β proportional to the stiffness matrix) was adopted. The results of this analysis allowed us to verify that the north arcade presents a similar behaviour to one obtained from the pushover analysis for the same seismic action, namely collapse mechanism and displacements (Figure 21c).

### 5.2. Discrete Element Model

**Figure 22:**
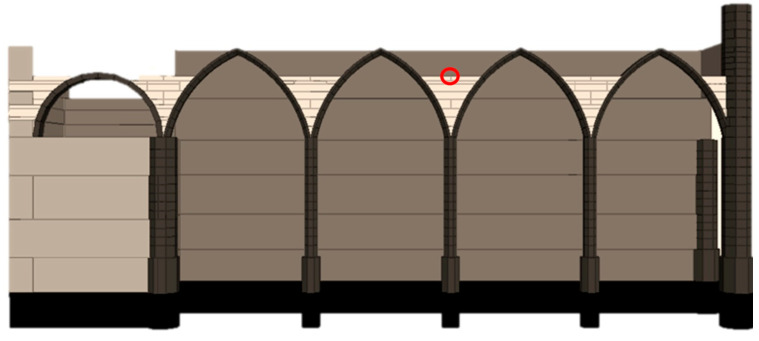
DEM model of the north arcade (control point highlighted in red).

For comparison of seismic analysis methods, a partial DEM model was also produced for the church of the Carmo Convent. The model was developed in 3DEC software [37]. Based on the scope of this work, only the north arcade was modelled, as it was identified as the most vulnerable part of the church based on the FEM analysis (Figure 22). This model contains 360 blocks, each with 6 degrees of freedom. The geometry was modelled in Rhino3D [12] and imported into 3DEC using a script produced by Itasca. After importing the geometry, the foundations were fixed, preventing translational movements. The edges of the blocks were then triangulated to increase the number of contacts between blocks and, therefore, improve the calculation of their interaction.

The material properties were defined according to those used in the FEM model to be able to perform the best comparison between the two modelling approaches. The density and Young’s modulus were set according to the values in Table 7. To apply the self-weight in 3DEC, gravity is “turned on”, and the program performs an automatic computation based on the density of the blocks. For this model, 10,000 cycles were run to obtain a negligible unbalanced force (1.0 × 10^−4^ N) and obtain the stability of the structure under its self-weight. Considering that in DEM, the blocks are distinct, material properties must also be defined for the interface between blocks. Thus, additional properties of joint normal stiffness, joint shear stiffness and friction angle are required. Joint shear stiffness was assumed to be 40% of the joint normal stiffness, according to the findings of previous studies [38]. The friction angle was defined based on experimental values found in the literature [39,40,41,42]. Finally, the joint tensile strength was assumed to be equal to that of the masonry in the FEM model (200 KPa), and the cohesion can be assumed as twice that value [38].

**Figure 23:**
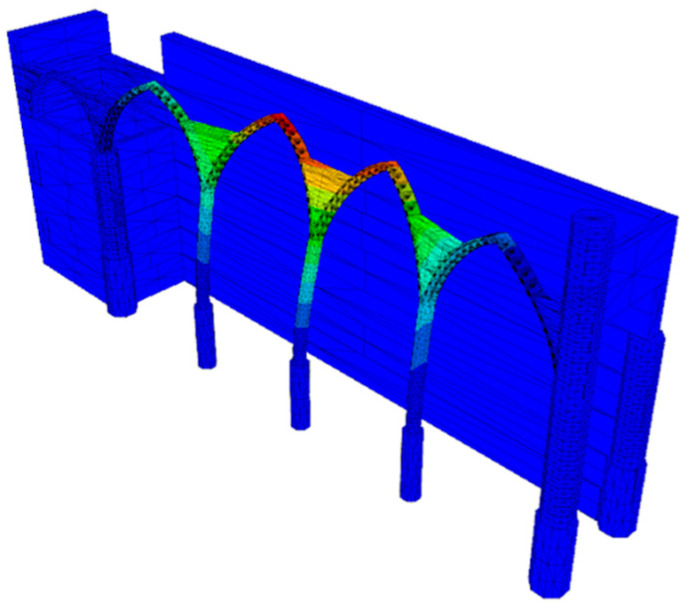
First mode of the north arcade in the transverse direction (DEM model).

A calibration of the joint normal stiffness was performed based on the first mode of the north arcade in the transverse direction (Table 8 and Figure 23), accounting for its highest contribution to the seismic behaviour. Next, the calibration was performed through iterative eigenvalue analyses to obtain a numerical frequency that matches the experimental value.

The whole structure is made of good-quality limestone masonry with thin mortar joints. Only the arcade filling has substantially different interfaces with thicker mortar joints. However, because of its location on the structure, it has little impact on the overall performance, and only a type of masonry was adopted.

**Figure 24:**
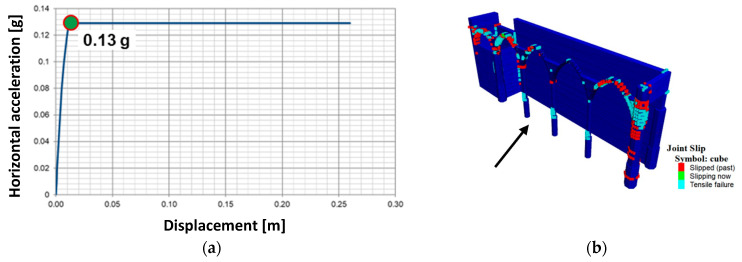
Pushover in the positive transverse direction for the DEM model: (**a**) capacity curve (see control point in Figure 18), (**b**) joint slip action in the structure at the maximum load (the arrow indicates the seismic action direction)..

In order to perform a pushover analysis, instead of applying a horizontal acceleration to the structure, which is not possible in 3DEC, a uniform distributed load was applied across the arcade, equal to the total inertial forces. In this way, this load pattern is not exactly proportional to the mass of each unit. Thus, it corresponds to a simplified method for applying the inertial forces caused by an earthquake. However, it allows for a reasonable approximation of a load factor that relates only to the mass of the arcade. The value obtained from the partial DEM model, equal to 0.13 g, is similar to the one from the FEM (0.10 g) (Figure 24). The displacements at failure are nearly identical for both analyses. Even if similarities in the response are to be expected as the two models use the same material properties, when applicable, it also validates the comparison of the damage mechanisms, namely the out-of-plane collapse of the north arcade (crack width for FEM and joint slip for DEM). The two numerical approaches present similar failure mechanisms, with the most severe damage concentrated in the arches closest to the walls and transept and at the columns close to the base, locations with high differences in stiffness between adjacent elements and the least ability for structural deformation.

Finally, regarding the expected seismic hazard of the church of Carmo Convent, a horizontal peak ground acceleration (PGA) of 0.22 g and 0.25 g are expected for an earthquake of Type 1 and 2, respectively (return period: 475 years; soil factor: 1.46; and soil type C, deep deposits of dense or medium-dense sand, gravel or stiff clay with thickness from several tens to hundreds of meters) [36,43]. Although the seismic capacity is lower than the seismic action, the confidence in the numerical results should be increased using other types of analysis, such as limit analysis and nonlinear dynamic analysis with time integration with different types of earthquakes for the full FEM model, to then perform seismic assessment based on limit states defined in terms of displacements. Moreover, it is recommended to evaluate the efficiency of strengthening techniques (for example, steel ties/cables, steel grid, glass roof, anchors, etc.) to improve the seismic performance of the arcades and the external walls of the nave. The stakeholder’s decision for the strengthening should take into account not only the level of the increase in the seismic capacity but also the impact caused on the monument. For example, it could be acceptable not to strengthen the building if the strengthening techniques to provide the required design seismic capacity cause a very high impact on the monument, changing its identity, or if strengthening reaches a seismic capacity lower than the one of design but causes a minor impact on the monument.

## 6. Conclusions

This paper aims to evaluate the effect of the underlying metro line and operating trains on the dynamic performance of the church ruins of the Carmo Convent, Lisbon, Portugal. Accounting for the assessment framework and threshold levels of particle velocities in international standards, monitoring acquisitions were performed with a geophone in three locations at the base of the structure, i.e., at the base of two columns and a corner of the north chapel. Accounting for the processed results, the level of induced ground-borne vibrations was several times lower than the maximum allowed, and thus, no risk of metro-induced ground-borne vibrations was identified for the monitored period, mainly due to the presence of the underlying metro station and the low speed of passing trains.

The evaluation of the dynamic behaviour of the structure for the seismic action was performed. The process in the current study navigated through various stages of investigation of performing an in situ visual inspection and NDT, allowing us to frame the damage level of the church; further calibrating the models for natural frequencies of the arcades; investigating the geometry of the arcade column interiors; updating the material properties and geometry; evaluating the application of the self-weight; and performing numerical analysis using the finite element method (FEM) and discrete element method (DEM). The visual inspection demonstrated that the main damage observed on the structure corresponds to biological growth and staining on the masonry. The morphology of the cross-section of the arcade columns was evaluated through GPR tests, which showed that these columns are likely composed of two types of masonry (external leaf and inner rubble masonry). The natural frequencies were estimated from dynamic identification tests based on ambient vibrations. These tests allowed for the estimation of five modes of the arcades, which ranged from 1.61 Hz to 3.91 Hz, and were used to calibrate the numerical models. Before performing the seismic analysis of the FEM model, the vertical loading of the self-weight was applied. First, the most conventional approach to apply the self-weight of the structure was adopted, i.e., application of the total self-weight to the entire geometry of the building in steps. This option does not accurately represent the procedure of constructing a masonry building and caused unrealistic initial damage in the FEM model, mainly in the arches. Thus, a phased analysis was performed, in which portions of the church and the respective self-weights were applied in phases. For this procedure, the initial damage caused by the self-weight loading was significantly reduced. The evaluation of the seismic behaviour was performed using pushover analyses, with horizontal load patterns proportional to the mass, aimed to evaluate the capacity of the structure and identify the most seismically vulnerable parts of the church. A nonlinear analysis with time integration was also carried out using a partial model of the north arcade. The seismic analysis of the updated FEM model allowed us to conclude that the north arcade is the most vulnerable element of the structure. The maximum horizontal acceleration for the north arcade is equal to 0.10 g and is associated with the out-of-plane collapse of the central columns and arches. The DEM model confirmed the collapse mechanisms and presented a similar capacity and displacement to the ones obtained from the FEM analysis. However, it is recommended to validate the results obtained in this study using other types of analysis, namely limit analysis with macroblocks and nonlinear dynamic analysis with time integration for the entire structure using different types of earthquakes. Finally, the efficiency of strengthening techniques should also be evaluated to improve the seismic capacity of the arcades and external walls of the nave (for example, steel ties/cables, steel grid, glass roof, anchors, etc.), in which their impact on the monument should also be considered.

## Figures and Tables

**Table 1:** Sonic tests results (average values).

Location	Young’s Modulus [GPa]	
	Direct Test	Indirect Test
Column A	25.9	4.6
Column B	19.3	4.9
Wall N1	16.4	-
Wall L	--	2.2
Wall T1	18.5	3.9
Wall T2	2.9	2.1
Wall T3	14.9	4.4
Wall C1	6.6	1.1
Wall C2	7.9	5.4
Buttress R1	10.9	4.2
Buttress B1	22.1	-
Façade FI	-	6.9
Façade FE	-	3.6

**Table 2:** Basic statistics in absolute particle velocities of setup in V2, during hours of no metro operation (01:00–06:30 am).

	Peak Velocity x-x [mm/s]	Peak Velocity y-y [mm/s]	Peak Velocity z-z [mm/s]
Max	0.0391	0.0590	0.0416
Min	0	0	0
Mean	0.0013	0.0015	0.0009
Median	0.0010	0.0011	0.0007
STD	0.0012	0.0017	0.0010
COV	0.9%	1.1%	1.1%

**Table 3:** Basic statistics in absolute particle velocities of setup in V1, during metro operational hours (06:00 pm to 01:00 am).

	Peak Velocity x-x [mm/s]	Peak Velocity y-y [mm/s]	Peak Velocity z-z [mm/s]
Max	0.0439	0.0359	0.0364
Min	0	0	0
Mean	0.0021	0.0020	0.0018
Median	0.0015	0.0015	0.0013
STD	0.0021	0.0018	0.0016
COV	1%	0.9%	0.9%

**Table 4:** Basic statistics in absolute particle velocities of setup in V2, during metro operational hours (07:00 pm to 01:00 am and 06:30 am to 09:00 am).

	Peak Velocity x-x [mm/s]	Peak Velocity y-y [mm/s]	Peak Velocity z-z [mm/s]
Max	0.0471	0.0772	0.0429
Min	0	0	0
Mean	0.0021	0.0026	0.0016
Median	0.0016	0.0019	0.0012
STD	0.0021	0.0026	0.0016
COV	1%	1%	1%

**Table 5:** Basic statistics in absolute particle velocities of setup in V3, during hours of no metro operation (11:00 am to 01:00 pm).

	Peak Velocity x-x [mm/s]	Peak Velocity y-y [mm/s]	Peak Velocity z-z [mm/s]
Max	0.0824	0.0636	0.1441
Min	0	0	0
Mean	0.0015	0.0017	0.0016
Median	0.0013	0.0014	0.0013
STD	0.0013	0.0015	0.0015
COV	0.9%	0.9%	0.9%

**Table 6:** Frequency calibration and attempts features for the longitudinal wall.

Experimental Frequency (Hz)	Numerical Frequency (Hz)	Attempts Calibration
5.7	1.83	E = 1.7 GPa
5.7	3.24	foundations level = 1.5 m, E = 5 GPa
5.7	5.0	foundations level = 3 m, E = 15 GPa
5.7	5.76	foundations level = 3 m, E = 16 GPa

**Table 7:** Material properties for the FEM before and after modal calibration and after phased sensitivity analysis.

Location	Young’s Modulus [GPa]		*v*	Density [kg/m^3^]	*f_t_* [MPa]		*f_c_* [MPa]		G_t_ [N/m]	G_c_ [kN/m]
	Prior	After			Prior	After	Prior	After	After	After
Arcade arches	35.0	35.0	0.2	2240	0.2	0.2	59	59	50	28
Arcade columns	35.0	35.0	0.2	2240	0.2	0.2	59	59	50	28
Arcade filling	2.9	2.4	0.2	2140	0.15	0.15	3.9	3.9	50	6.3
Longitudinal walls	18.0	18.0	0.2	2140	0.2	0.2	30	30	50	25
Chapels filling	1.0	1.0	0.2	2000	0.1	0.1	1.7	1.7	25	2.7
Transverse walls of the chapels and main façade	2.9	2.9	0.2	2140	0.15	0.15	4.8	4.8	50	7.7

**Table 8:** Material properties for the FEM model after calibration.

Joint Normal Stiffness (MPa/m)	Joint Shear Stiffness (MPa/m)	Friction Angle	Cohesion	Joint Tensile Stiffness (MPa/m)
2200	880	35	400	200

## Data Availability

The data is not available in any online database.
